# A novel *BCHE* frameshift mutation in a Chinese woman with butyrylcholinesterase deficiency: A case report and literature review

**DOI:** 10.1097/MD.0000000000039976

**Published:** 2024-10-04

**Authors:** Jiantao Zeng, Dan Yang, Tao Dai, Jun Xia, Zhaobin Zheng

**Affiliations:** a Department of Laboratory Medicine, People’s Hospital of Changshou Chongqing, Chongqing, China; b Department of Nephrology, Chongqing Changshou District Hospital of Traditional Chinese Medicine, Chongqing, China; c Department of Cardiovascular, People’s Hospital of Changshou Chongqing, Chongqing, China.

**Keywords:** *BCHE* gene, butyrylcholinesterase deficiency, frameshift mutation, silent phenotype

## Abstract

**Rationale:**

Congenital butyrylcholinesterase deficiency (BCHED) is a rare autosomal recessive genetic disorder caused by a pathogenic mutation in the *BCHE* gene. Patients with BCHED may experience prolonged apnea or even death after the application of drugs such as succinylcholine. We aimed to identify the genetic basis of disease in a patient presenting with butyrylcholinesterase deficiency in order to confirm the diagnosis, expand *BCHE* gene mutation spectrum, and elucidate potential genotype-phenotype associations to inform management.

**Patient concerns:**

A 51-year-old woman presented with “vague pain in the upper and middle abdomen.” Her serum cholinesterase level was 211 U/L (reference value 4000–13,000 U/L). Other laboratory findings were normal. Genetic analysis revealed compound heterozygous mutations in *BCHE* gene, which was considered pathogenic in this case.

**Diagnoses:**

The patient presented with low serum cholinesterase levels, which excluded common causes such as liver disease, drug toxicity, and chronic illness. Whole exon examination revealed compound heterozygous mutations in the *BCHE* gene; thus, the patient was diagnosed with congenital BCHED.

**Interventions:**

Gastroscopy without succinylcholine or mivacurium chloride was recommended. The gastroscopy results were “gastric polyps,” and gastroscopic “polypectomy” was performed. The patient was advised to avoid succinylcholine use.

**Outcomes:**

The patient’s serum cholinesterase level was reviewed 3 months later, and the result was 215 U/L. Double heterozygous mutations are the cause of BChE deficiency of this woman in this study, including a novel mutation NM_000055.4: c.666_669del (p.Phe223Glufs*38). A review of the literature reveals considerable variation in the hotspot variants of the *BCHE* gene across different populations. The Chinese population displays a higher prevalence of the silent type, which is more sensitive to anesthetics such as succinylcholine.

**Lessons:**

Clinical manifestations of congenital BCHED were not significant. This study avoided a potential anesthetic accident, and the novel variant enriched the *BCHE* gene mutation spectrum.

## 1. Introduction

Butyrylcholinesterase (BChE) and acetylcholinesterase (AChE) are 2 types of human cholinesterases. AChE is predominantly found in the synaptic gaps of the cholinergic nerve endings. BChE is mainly produced in the liver and distributed throughout all tissues. AChE hydrolyzes acetylcholine, thereby terminating its activity. Unlike AChE, BChE is an ester hydrolase that hydrolyzes certain short-acting neuromuscular blockers such as succinylcholine and mivacurium chloride.^[1]^

Congenital BChE deficiency (BCHED; OMIM #617936) is a rare autosomal recessive genetic disorder caused by a pathogenic mutation in *BCHE* gene. Patients with BCHED are usually not life-threatening; however, because of their high sensitivity to drugs, such as muscle relaxants, they may experience prolonged apnea or even death after the application of similar drugs.^[2,3]^

Patients with BCHED are typically asymptomatic and are often only detected following anesthesia or electroconvulsive therapy,^[4–6]^ but intellectual disability has occasionally been reported in children with congenital BCHED.^[7]^

This article provides clinical information about a patient with BChE deficiency. Congenital BCHED was confirmed by genetic testing, and anesthetic accidents were avoided. A new *BCHE* variant was identified using whole-exome sequencing, further extending the phenotypic spectrum of *BCHE* variants.

We conducted a comprehensive review of the literature and identified a correlation between *BCHE* variants and serum cholinesterase activity. Additionally, we preliminarily analyzed common *BCHE* variants in different ethnic populations.

## 2. Case presentation

### 2.1. History of present illness

A 51-year-old woman presented with “vague pain in the upper and middle abdomen.” After regular biochemical examinations, we observed an extremely low level of BChE activity in the liver function tests. BChE activity was measured using a colorimetric butyrylthiocholine assay with a BChE kit on a Beckman 5800 chemistry autoanalyzer. The results showed that serum cholinesterase level was 211 U/L (reference range: 4000–13,000 U/L). The patient completed other laboratory tests, with no significant abnormal results.

### 2.2. Physical examination

The patient was 51 years old, 155 cm long, and weighed 50 kg. She was conscious, with a moderate nutritional status, no yellowing of the skin, and no palpable lymph nodes. Breath sounds were clear in both lungs, with no dry or wet rale. Her heart rate was 85 beats/min, and no abnormal murmurs were detected on auscultation. The abdomen was soft. The liver and spleen were not palpable below their ribs. No abnormalities were observed in the spine, extremities, anus, or the external genitalia. There had no history of hypertension, diabetes mellitus, cardiac disease, hepatitis, or renal disease.

### 2.3. Laboratory examinations

The results of these laboratory studies are presented in Table 1. BChE activity was significantly reduced, whereas alanine aminotransferase (ALT), γ-glutamyltransferase (GGT), and cholesterol (CHOL) levels were mildly elevated. All other test results were within the normal ranges. We conducted an analysis to evaluate hepatitis markers and antibodies against liver disease. The analysis revealed that anti-hepatic and renal microsomal antibodies, anti-soluble liver antigen antibodies, and anti-hepatocyte cytosol antibodies were negative. Additionally, the hepatitis A virus (HAV) IgM, hepatitis B virus surface antigen, hepatitis C virus IgG, and hepatitis E virus IgM tests were negative. Based on these results, it can be inferred that the low serum cholinesterase levels were not caused by liver disease.

**Table 1:** The laboratory test results.

Examination items	Results	Normal range
BChE	211.0 U/L ↓	4000–13,000 U/L
ALT	49.9 U/L ↑	7–40 U/L
AST	34.2 U/L	13–35 U/L
GGT	58.9 U/L ↑	7–45 U/L
ALP	89.8 U/L	50–135 U/L
TP	76.9 g/L	65–85 g/L
ALB	46.9 g/L	35–55 g/L
TBIL	10.4 µmol/L	0–21 µmol/L
CHOL	5.4 mmol/L ↑	2.3–5.2 mmol/L
TG	1.6 mmol/L	0.5–1.7 mmol/L
Bu	3.9 mmol/L	2.6–7.5 mmol/L
Cre	56.3 µmol/L	41–73 µmol/L

↑ = above reference range, ↓ = below reference range, ALB = albumin, ALP = alkaline phosphatase, ALT = alanine aminotransferase, AST = aspartate aminotransferase, BChE = butyrylcholinesterase, Bu = blood urea nitrogen, CHOL = cholesterol, Cre = creatinine, GGT = γ-glutamyltransferase, TBIL = total bilirubin, TG = triglyceride, TP = total protein.

### 2.4. Results of genetic analysis

Based on the patient’s physical examination and laboratory results, we considered that the patient might have congenital BChE deficiency and performed whole-exome sequencing.

**Figure 1.**
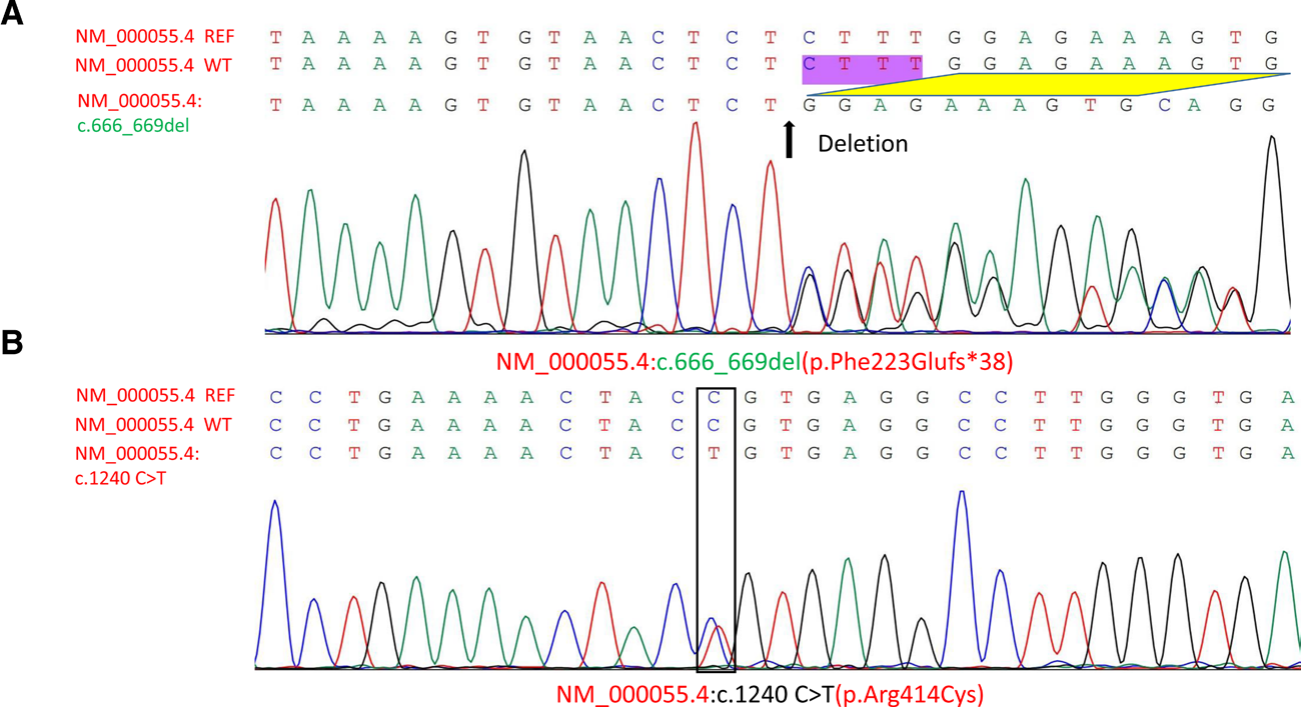
Sanger sequencing of the *BCHE* gene mutations. (A) NM_000055.4: c.666_669del (p.Phe223Glufs*38) frameshift heterozygous mutation in exon2. (B) NM_000055.4:c.1240C>T (p.Arg414Cys) heterozygous mutation in exon2. (A) The 3 sequences are presented in order, beginning with the NM_000055.4 (*BCHE*) gene reference sequence, followed by the NM_000055.4 (*BCHE*) wild-type sequence, and concluding with the NM_000055.4 (*BCHE*):c.666_669del variant sequence. Black arrow and “Deletion” indicate the locations of base deletions. The purple background labeled “CTTT” indicates the missing base of the NM_000055.4:c.666_669del variant. Yellow diamonds indicate sequence comparison between the *BCHE* wild-type sequence and the NM_000055.4: c.666_669del variant sequence. (B) The 3 sequences are presented in order, beginning with the NM_000055.4 (*BCHE*) gene reference sequence, followed by the NM_000055..4 (*BCHE*) wild-type sequence, and concluding with the NM_000055..4 (*BCHE*):c.1240 C > T (p.Arg414Cys) variant sequence. The black box indicates that the patient has a NM_000055.4:c.1240 C > T (p.Arg414Cys) variant in the *BCHE* gene.

Whole-exome sequencing revealed 2 variants of *BCHE* associated with BChE deficiency. The 2 variants were NM_000055.4:c.666_669del (p.Phe223Glufs*38) and NM_000055.4:c.1240C>T (p.Arg414Cys) heterozygous mutations; Sanger sequencing confirmed these deletions (Fig. 1A) and point mutations (Fig. 1B). Therefore, the patient was diagnosed with congenital BCHED.

### 2.5. Interventions and outcomes

Considering that patients with BChE deficiency may experience adverse reactions to muscarinic agents, gastroscopy without succinylcholine, or mivacurium chloride is recommended. The gastroscopy results were “gastric polyps,” and gastroscopic “polypectomy” was performed. The patient recovered well after the surgery and had no adverse reactions. The patient’s serum cholinesterase level was reviewed 3 months later, and the result was 215 U/L (reference range: 4000–13,000 U/L).

### 2.6. Literature review

The NM_000055.4:c.666_669del (p.Phe223Glufs*38) deletion has not been reported in the literature and is a novel variant of *BCHE* gene. The ClinVar database reports a deletion at a comparable location: NM_000055.4:c.666_667del (p.Phe223Trpfs*10). This delete variant lacked 2 bases, resulting in a change from phenylalanine to tryptophan at codon 223, and premature termination of the reading frame at codon 232.

To investigate the relationship between *BCHE* variants, BChE activity and ethnicity, we searched the PubMed database using the keyword “butyrylcholinesterase deficiency.” The literature containing data on *BCHE* genetic testing was also screened. A total of 24 papers were included, and the results are shown in Supplementary S1, Supplemental Digital Content, http://links.lww.com/MD/N682.

## 3. Discussion

BChE is mainly synthesized by the liver and distributed throughout the body, especially in the liver, lungs, and brain. Therefore, damage to organs, such as the liver, can decrease BChE levels.^[8]^ The most common clinical decrease in serum cholinesterase levels occurs in organophosphate pesticide poisoning, severe liver disease, and severe malnutrition, whereas congenital BChE deficiency is rare and requires genetic testing for diagnosis.^[9,10]^

*BCHE* gene is located on chromosome 3 (3q26.1-q26.2) and consists of 4 exons. At present, the ClinVar database contains more than 150 *BCHE* variants, most of which are point mutations and a few are deletions or insertions. Congenital BCHED is a rare autosomal recessive genetic disorder. Therefore, homozygous mutations had a greater impact on BChE levels.^[11]^ Previous literature has reported that heterozygous mutations alone have little effect on serum cholinesterase levels.^[12]^ However, our study demonstrated that compound heterozygous mutations can also lead to disease.

Current research shows that the incidence rate of congenital BCHED and serum cholinesterase levels are quite different in different populations. The most important variants were atypical variants, Kalow variants (BChE ≤30% of normal levels), fluoride variants, and silent variants (BChE ≤10% of normal levels). In the White population, the atypical variants and Kalow variants were the most common. Silent variants are more prevalent in Asia.^[9]^

At present, no large-scale cohort studies related to congenital BCHED in China have been reported in the literature; therefore, the type and prevalence of *BCHE* variants in the Chinese population are still unclear. However, the current case report data (Supplementary S1, Supplemental Digital Content, http://links.lww.com/MD/N682) show that the common type of *BCHE* gene point mutation in Chinese individuals is NM_000055.4:c.1240C>T (p.Arg414Cys) heterozygous mutation, and the main insertion-type change was NM_000055.4:c.401dup (p.Asn134Lysfs*24), both of which are prone to exhibiting silent BCHED. In this case, the mutation was a compound heterozygous mutation, which may have resulted in the patient presenting with typical silent BCHED.

The incidence of *BCHE* homozygous mutations varies significantly among different populations. Previous studies have reported that the incidence of *BCHE* heterozygous mutations is 2/1000,^[13]^ and the incidence of homozygous mutations ranges from 2/10,000 to 5/10,000.^[14,15]^ However, in the Vysya community in India, a study randomly tested 221 residents in the area, with 9 cases of homozygous *BCHE* mutations.^[16]^ This percentage was significantly higher than that in other regions.

At present, research on the physiological function of serum BChE is not comprehensive and patients with BCHED usually have no clinical symptoms. However, it has been found that after using muscle relaxants such as succinylcholine or mivacurium, patients with BCHED are prone to prolonged neuromuscular blockade or even respiratory arrest. This indicated that BChE plays an important role in the metabolism of muscle relaxants. According to recent reports, different mutant phenotypes exhibit inconsistent adverse reactions to muscle relaxants. There have been no reports of adverse reactions to muscle relaxants in the K-type homozygous mutant population, whereas more cases have been reported in the silent variant population.^[17]^ Therefore, it is necessary to pay attention to preoperative *BCHE* gene testing in anesthesia patients with BCHED.

While this case report expands the *BCHE* mutation spectrum, our study has some limitations. One such limitation was the inability to obtain complete sequencing data of the patient’s family. Additionally, our analysis did not include in vitro studies to directly assess effects of this novel mutation on enzyme function.

## 4. Conclusions

Overall, the patient had congenital BChE deficiency due to compound heterozygous mutations, with the NM_000055.4:c.666_669del (p.Phe223Glufs*38) deletion being the first reported mutation. Thus, this study avoided potential anesthesia accidents and helped further expanded the *BCHE* mutation spectrum. This case and literature review augments our comprehension of the phenotypic heterogeneity associated with *BCHE* mutations, and provide a foundation for future studies of potential genotype-phenotype correlations involving mutations in this gene.

## Author contributions

**Formal analysis:** Jiantao Zeng.

**Resources:** Jiantao Zeng.

**Writing – original draft:** Jiantao Zeng, Dan Yang.

**Writing – review & editing:** Jiantao Zeng, Dan Yang, Tao Dai, Jun Xia, Zhaobin Zheng.

**Investigation:** Dan Yang.

**Supervision:** Tao Dai, Jun Xia.

## Supplementary Material


